# Temporal Coordination in Mother–Infant Vocal Interaction: A Cross-Cultural Comparison

**DOI:** 10.3389/fpsyg.2019.02374

**Published:** 2019-11-08

**Authors:** Lama K. Farran, Hyunjoo Yoo, Chia-Cheng Lee, Dale D. Bowman, D. Kimbrough Oller

**Affiliations:** ^1^Communication Sciences and Disorders, University of West Georgia, Carrollton, GA, United States; ^2^Department of Communicative Disorders, College of Arts and Sciences, University of Alabama, Tuscaloosa, AL, United States; ^3^Department of Speech and Hearing Sciences, Portland State University, Portland, OR, United States; ^4^School of Communication Sciences and Disorders, University of Memphis, Memphis, TN, United States

**Keywords:** vocal turn-taking, cross-cultural language development, vocal development, vocal coordination, temporal synchrony, mother–infant interaction, niche construction, cross-cultural psycholinguistics

## Abstract

Temporal coordination of vocal exchanges between mothers and their infants emerges from a developmental process that relies on the ability of communication partners to co-coordinate and predict each other’s turns. Consequently, the partners engage in communicative niche construction that forms a foundation for language in human infancy. While robust universals in vocal turn-taking have been found, differences in the timing of maternal and infant vocalizations have also been reported across cultures. In this study, we examine the temporal structure of vocal interactions in 38 mother–infant dyads in the first two years across two cultures—American and Lebanese—by studying observed and randomized distributions of vocalizations, focusing on both gaps and overlaps in naturalistic 10-min vocal interactions. We conducted a series of simulations using Kolmogorov–Smirnov (K–S) tests to examine whether the observed responsivity patterns differed from randomly generated simulations of responsivity patterns in both Arabic and English for mothers responding to infants and for infants responding to mothers. Results revealed that both mothers and infants engaged in conversational alternation, with mothers acting similarly across cultures. By contrast, significant differences were observed in the timing of infant responses to maternal utterances, with the Lebanese infants’ tendency to cluster their responses in the first half-second after the offset of the Lebanese mothers’ utterances to a greater extent than their American counterparts. We speculate that the results may be due to potential phonotactic differences between Arabic and English and/or to differing child-rearing practices across Lebanese and American cultures. The findings may have implications for early identification of developmental disorders such as autism spectrum disorders within and across cultures.

## Introduction

### Background on Parent–Infant Interaction

Conversational exchanges between mothers and their young children have been claimed to be ubiquitous across languages and cultures (Bateson, 1975; Stern et al., 1975; Fernald et al., 1989; Papoušek and Papoušek, 1989; Lavelli and Fogel, 2013) and a hallmark of human sociality (Levinson, 2016). These affectively charged interactions are temporally structured and seem to be shared across species (Takahashi et al., 2013), reflecting phylogenetic origins. As such, they constitute the affordances (Trevarthen, 1979; Loveland, 1991) within which the mother–infant dyad co-coordinates, recruits, and fine-tunes its vocalizations as it engages in communicative niche construction (Odling-Smee, 1988; West et al., 1988), paving the way for the development of language in human infancy.

The temporal structure of early mother-child vocal interactions has been the focus of several recent studies (e.g., Jaffe et al., 2001; Gros-Louis et al., 2006; Kondaurova et al., 2016), suggesting exchanges characterized by precise gaps and overlaps between the utterances of communication partners (Levinson, 2016) across cultures (Keller et al., 1999). The evidence indicates ontogenetic, dyad-specific patterns of mutually co-coordinated, bidirectional attunement from mother and infant (Ko et al., 2015), reflecting primary intersubjectivity (Trevarthen, 1977), which progressively develops into predictable interactions that form the basis for the development of social communication in infancy (Papoušek and Papoušek, 1989).

Research has suggested that from around 3 to 4 months of age, infants act as communication partners capable of predicting accurately and with remarkable ease when a conversational turn will end and another turn will begin; the infant’s predictions are thought to be based on pragmatic, semantic, and syntactic factors (Gratier et al., 2015) as well as suprasegmental (stress, pitch, intonation) aspects of caregiver speech. By around 5–6 months of age, infants have been reported to engage in protoconversations (Bates et al., 1979) whereby they convey their intentions and engage in social communicative exchanges, still long before they become language users. While we recognize that these exchanges are essentially multimodal in nature, we focus on the vocal modality only, as the primary channel for communication during early interactions between mothers and their infants (Van Egeren et al., 2001). Furthermore, we hypothesize that while early conversational turn-taking is very widespread across cultures (Stivers et al., 2009), it may manifest cross-cultural variation as a function of differences in the social use and structure of the languages in which interactions occur (Lupyan and Dale, 2010; Farran et al., 2016). In fact, while quantification has been scanty, some studies have reported major differences in the amount of parent-infant interaction occurring across cultures, with much lower rates in some low-technology societies (Ochs and Schieffelin, 1984; Correa-Chávez and Rogoff, 2009; Cristia et al., 2017). Moreover, there is emerging evidence to suggest reduced amounts of interaction occurring between mothers and their infants and young children who may be at-risk for, or diagnosed with, autism spectrum disorder (ASD) (Warlaumont et al., 2010; Northrup and Iverson, 2015), which appear associated with derailment of early speech and language development. Importantly, the majority of the studies of parent–infant vocal interaction have been confined to western cultures. This cultural bias in available research suggests a need for comparative empirical studies of mechanisms that underlie cultural differences in typically developing infants and their mothers. Such work may also lay the foundations for examining vocal coordination between at-risk infants and their mothers and designing early intervention studies aimed at understanding the range of young children’s vocal–including disordered–development, within and across cultures.

A growing body of research suggests that while both mothers and infants may contribute to early communicative exchanges (Cohn and Tronick, 1988; Feldman, 2007; Sung et al., 2013; Bigelow and Power, 2014; Gros-Louis et al., 2014), mothers seem to play a more influential role than their infants, often driving the conversational flow and ensuring the maintenance of contingent conversational turns (Jaffe et al., 2001; Yoo et al., 2018) and regular conversational turns (Jaffe et al., 2001) that tend to become more refined with experience (Gratier et al., 2015). The amount of interaction is correlated with positive outcomes in attachment, speech, and language development (Goldstein and Schwade, 2008). Recent evidence also suggests that infants, even as young as 8–21 weeks, may play an active role in initiating interactions and maintaining conversational turns (Dominguez et al., 2016). Likewise, Mothers play a critical role in early vocal interaction. Yoo et al. (2018) have shown that mothers tended to alternate with infant speech-like Vocalizations from the beginning of life, suggesting that they intuitively facilitate protoconversation, while they produce overlapping vocalizations that tend to soothe a crying infant.

Examining the temporal intervals between mothers’ and infants’ vocalizations is a particularly fundamental realm for understanding early typical (Jasnow and Feldstein, 1986; Beebe and Lachmann, 1988; Stivers et al., 2009) and atypical (Warlaumont et al., 2010, 2014; Smith and McMurray, 2018) development within and across cultures. Temporal vocal coordination has been suggested to be crucial in uncovering developmental anomalies characteristic of, for example, ASD in the first two years, thereby potentially informing early detection and intervention (Northrup and Iverson, 2015).

This type of coordination is contingent upon learning by both conversational partners (Goldstein et al., 2003, 2009). The infant must attune to the temporal structure of conversational turns by adapting to the vocal input pattern of the mother. Similarly, the mother must learn her child’s vocal cues and adapt her input to suit her child’s language and developmental level. Evidently, variation in one conversational partner’s vocalization pattern is shaped by variation in the other conversational partner’s vocalization pattern, reflecting co-regulation—the coupling of interactions that presumably becomes more coordinated with development (Fogel, 1977; Kaye and Fogel, 1980). Studying the vocalization patterns of both conversational partners is thus necessary to help determine the extent of temporal coordination between mothers and their infants as a precursor to language development.

The back-and-forth temporal coordination between mother and infant has been the focus of numerous studies (e.g., Bateson, 1975; Snow, 1977; Ginsburg and Kilbourne, 1988), and the results have suggested a predictable temporal structure of maternal vocalizations usually occurring within the first second after the termination of infant vocalizations. The pertinent studies have, however, usually not quantified both gaps (indicating alternation between mothers’ and infants’ utterances) and overlaps (indicating non-alternation between mothers’ and infants’ utterances). Moreover, many studies have had very small samples, consisting usually of 1–3 children.

In a recent longitudinal study, Hilbrink et al. (2015) presented data that were, to their knowledge, the first to address both gaps and overlaps in vocal responses of infants to mothers and mothers to infants. The study assessed the temporal structure of conversational turns in 12 infants ages 3, 4, 5, 9, 12, and 18 months and their mothers. Based on a permutation test, the authors reported that infants showed systematic vocal responsivity to mother utterances even at 3 months. Accordingly, they reported changes in infant responsivity across time: infants’ responses were slower at 9 and 12 months than at the other ages, a finding the authors interpreted as owing to the infants’ growing awareness of words in their ambient language.

Subsequent work by Yoo et al. (2018) examining vocal development in 12 infants at 0, 1, and 3 months along with their parents, also addressed gaps and overlaps in maternal vocal responses, but did so in several ways that differed from Hilbrink et al. (2015). First, the effort by Yoo et al. (2018) differentiated maternal responses to speech-like infant vocalizations (termed “protophones” after Oller, 2000) from cry/distress vocalizations, while the Hilbrink et al. (2015) effort had considered maternal responses to all infant sounds (except involuntary [vegetative] sounds), thus including responses to cries, on the grounds that “mothers often treated these sounds as communicative (p. 5).” Yoo et al. (2018) found, however, that mothers’ responses tended to overlap with cries, while alternating (showing gaps) in response to protophones. The authors argued that the differentiation was functional, in that mothers seemed to be attempting to soothe infants who were crying, and thus often spoke comfortingly while the infants cried, whereas they treated the protophones as potential material for conversation, and thus tended to wait for infants to complete their conversational turns before speaking themselves. In addition, the two studies differed in that Hilbrink et al. (2015) presented the data on overlap and gap responses separately, while Yoo et al. (2018) presented distributions where all the data were assessed together. The latter approach has the advantage of implying no assumption that either mothers or infants intend to produce overlapping and alternating responses in categorically different ways. Instead the latter approach allows direct portrayal of a continuum of responsivity with degrees of overlap or alternation illustrated in a combined distribution.

The present work follows trends of prior research, with a few methodological adjustments as will be seen below. Furthermore, we are intrigued by the need to examine temporal coordination and timing of vocalizations in mother-infant interaction across different cultures (Keller et al., 1999; Stivers et al., 2009; Kärtner et al., 2010). It seems likely that there may be important variations across cultures in the timing of interactions, just as it has been claimed that there are important differences in the raw amount of vocal interaction that occurs with infants across cultures (Dixon et al., 1981; Ochs and Schieffelin, 1984; Richman et al., 1992; Lieven, 1994; Whaley et al., 2002; Le Vine, 2004; Cristia et al., 2017). Our effort has been motivated in part by stark differences in the syllable structure and lexical stress patterns across Arabic and English, in addition to cultural differences that we alluded to in the previous section and possible differences in child-rearing practices that characterize the Lebanese and American cultures. We turn to these differences next.

### Background on the Structure of the Arabic Language

Arabic is a Semitic language, spoken by approximately 300 million people worldwide, and characterized by diglossia, which is the co-existence of two forms of the language that work together to serve different, yet complementary sets of purposes: (1) Modern Standard Arabic (MSA) or “fusha,” which is a derivative of classic Arabic and the variant used for formal education, mainly for reading and writing; and (2) spoken Arabic known as “Amiya” with many varieties (vernaculars or dialects) acquired by native speakers as their first language.

Arabic consists of 28 consonants, with consonant-vowel (CV) as the minimal syllable. Importantly, Arabic stress may vary as a function of the spoken vernacular. It is non-phonemic (Holes, 2004, p. 62), non-disinctive (Kager, 2009, p. 344) and predictable, though dialect-dependent (Saiegh-Haddad and Henkin-Roitfarb, 2014). To illustrate, Aquil (2011) reviewed Cairene (Egyptian) Arabic and posited that “stress in Cairene Arabic is almost entirely predictable” (p.5). In the case of Lebanese Arabic (one of the target languages in this study), it is presumable that word stress is similarly predictable, although this awaits empirical substantiation.

In addition to the identified differences in language structure, it appears that differences in vocal coordination may be based on different views within the Lebanese and American cultures regarding whether, and degree to which, infants are conceptualized and treated as communicative partners (Keller et al., 2007) and whether they are expected to play an active role in shaping communicative exchanges with their mothers. For research on vocal interaction in the Lebanese society, families or kin groups can reasonably be thought to constitute the appropriate unit of analysis, consistent with the tenets of a collectivist culture. By contrast, the infant or the mother-infant dyad is treated as the appropriate unit of analysis within individualistic cultures such as the US (Bornstein et al., 1991; Keller et al., 2007). These differences in roles, namely focusing on the individual versus the group within societies, may impact the frequency and quality of early vocal interaction in the target cultures. Thus the availability of recorded data on mother-infant interaction in Lebanese and American pairs provides a useful starting point in evaluation of possible cultural effects in mother-infant interaction timing.

Our approach is based on dynamical systems theory (DST; Thelen and Smith, 1994), which conceptualizes early language development as an emergent phenomenon, where the history of interactions influences developmental processes and their constraining functions (Deacon, 2010). Central to DST is the understanding of how patterns relate to the co-regulation between constituents of the system, namely members of the dyad—mother and baby. Relevant to this study, the (vocal) behavior of one member (e.g., the infant) acts to constrain the multiple actions and behaviors of the other member (e.g., the mother), resulting in a complex system that organizes and re-organizes itself into *attractors* (macrolevel, stable patterns) that form the basis for recognizable, dyad-specific patterns of behavior. Thus, change occurs at the *microlevel*, and entails an iterative cycling in infant and mother vocal behavior as both dyad members shift from one co-coordinated communicative exchange to another. Each instance of a given attractor that occurs over time has a similar pattern of vocalizations, albeit different from that of the previous instance of vocalizations pertaining to this same attractor, culminating in within-attractor variability (semi-stable patterns of vocal behaviors). Change also occurs at the *macrolevel* and consists of co-occurring microlevel changes reflecting qualitative, nuanced differences in system organization that emerge from previous microlevel changes within attractors, and shifts are often perceived to be transitions in vocal coordination (for example, from less coordinated to more coordinated). The mother-infant dyads may tend toward tighter coupling of vocalizations as they co-construct the language niche for the two of them and for their communities. Presumably, this pattern would be disturbed in the case of atypical coordination seen in atypical development (e.g., ASD), since disruption in the initial state of back-and-forth vocal exchanges forms the basis upon which change at the microlevel occurs, which then becomes the raw material for macrolevel changes that mother–infant dyads use as they co-construct the language niche in ASD. Our aim in the present study includes examining the structure of mother-infant vocal exchanges, as these hold the potential to inform us regarding the dynamics of the system in both typical and atypical infants across development. We propose that possible developmental trajectories become essentially reliant on contextual factors, with ambient language playing a potentially important constraining role, even before infants produce symbols (Bates et al., 1979). Culture and language thus participate in a ratcheting pattern of niche construction (Deacon, 1997, 2010) with early vocal interaction playing a central role in language emergence.

### Purpose of the Current Study

The present study aims at examining the extent to which vocal exchanges between mothers and infants are reciprocally structured across Lebanese-Arabic and American-English dyads. We addressed the following research questions by evaluating combined distributions of both overlaps and alternations of vocal responses by mothers and infants:

(1) What is the timing of mothers’ vocalizations in response to their infants’ vocalizations within and across cultures?
(2) What is the timing of infants’ vocalizations in response to their mothers’ vocalizations within and across cultures?
(3) Are patterns of conversational turns different across the cultures?

## Materials and Methods

### Selection of Participants

The participants were 0-24-month-old infants and their mothers (the same sample as in Farran et al., 2016). The Lebanese sample consisted of 19 Lebanese Arabic-speaking mother-infant dyads (12 male and 7 female) recruited from two private and two public pediatric clinics in Lebanon. They came from low-mid SES backgrounds. The American sample consisted of 19 American English-speaking mother-infant dyads (9 male and 10 female) recruited originally in two University of Memphis longitudinal studies on infant vocal development. They came from low-mid to mid SES backgrounds (see Table 1). Infants from both cultures were typically developing and matched on maternal education, age, and gender.

**TABLE 1:** Participant characteristics for infants in the Lebanese and American samples.

**Arabic Group**		**English Group**	
	
**Infant ID**	**Age in Months**	**Infant ID**	**Age in Months**
A1	0	E4	1
A4	5	E13	5
A5	6	E16	6
A6	6	E19	6
A7	6	E18	6
A11	8	E2	8
A13	9	E5	9
A14	10	E7	10
A17	10	E17	10
A18	11	E14	11
A19	12	E15	12
A2	12	E9	12
A3	13	E6	12
A8	16	E12	16
A9	17	E3	16
A12	18	E1	18
A10	21	E10	20
A15	21	E8	21
A16	24	E11	24

### Procedure

Briefly we summarize here the procedure detailed in the prior article (Farran et al., 2016). We recorded 10-min samples of mother–infant interactions. For the Lebanese recordings, we asked the mothers to interact freely with their children as they normally did in the home. The recording equipment was high fidelity and the microphone was placed as near the interacting pair as possible but out of reach of the infant. The American recordings were drawn from an archive of longitudinal research at the University of Memphis. Samples had been digitally recorded for audio using wireless microphones worn on both infant and mother in a laboratory designed to resemble a child’s playroom. To enroll in the study, a written informed consent was obtained from all adult participants and parents of non-adult participants.

### The Coding Plan and Software

For the Lebanese recordings, the mothers’ utterances were coded in PRAAT (Boersma and Weenink, 2018), an acoustic analysis system available as on-line freeware that allows coders to view waveform and spectrographic displays in real-time, place cursors on the screen to indicate onset and offset of vocalizations, and determine the locations in time and durations of each parent and infant utterance. For the American recordings, we coded mothers’ and infants’ utterances using AACT (Action Analysis Coding and Training by Delgado et al., 2010) with audio represented in TF32 (Milenkovic, 2015). AACT allowed convenient determination of utterance boundaries and durations. For both Lebanese and American samples, coders identified the onset and offset of each utterance using a breath-group criterion (i.e., one utterance per breath group), as recommended by Lynch et al. (1995). We examined both parent responses to infants and infant responses to parents. All coded PRAAT files were converted into AACT coding files for further analysis.

Following the coding scheme of Farran et al. (2016), maternal utterances were characterized as infant-directed speech (IDS) if they were directed to the infant. A maternal utterance was considered to be a response to a baby utterance only if it was an instance of IDS. Similarly, an infant utterance, which consisted of protophones (speech-like sounds), was considered a response to a parent utterance only if it followed a maternal IDS utterance. Therefore, we conceptualized each data point to consist of a pair of utterances—an infant protophone and a maternal IDS utterance.

The first and third authors completed coding for this study. The first author was the main coder and is a speaker of Arabic as a primary language, English as a second language, and French as a third language. The third author, who served as the reliability coder, is a speaker of Mandarin as a primary language, Southern Min as a second language, and English as a third language. Both coders were trained extensively on the coding scheme and underwent two rounds of training in cursor placement under the supervision of the last author (with PRAAT), each coding a total of 38 sessions (19 sessions in Arabic and 19 sessions in English) independently. Intercoder reliability was computed using Intraclass Correlation (ICC), which resulted in a session-level reliability rating of Optimal-Excellent on both utterances per minute and seconds per minute (see Farran et al., 2016).

### Definition of Response Interval

**FIGURE 1:**
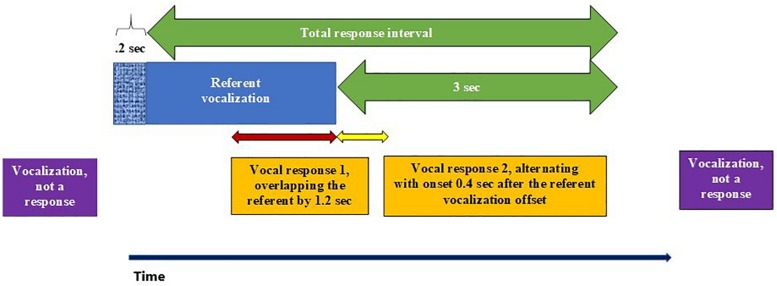
Definitions of responses. The blue, purple, and orange blocks represent either mother or infant vocalizations for either of two types of analysis (i.e., infant responses to mother or vice versa). The blue block indicates a referent vocalization, either by the mother or the infant. Purple and orange blocks indicate potentially responsive vocalizations of the other speaker, which are judged to constitute responses if and only if their start times are within the Total Response Interval (green arrow). Orange blocks do correspond to utterances beginning within the Total Response Interval, while purple blocks correspond to utterances that do not begin within the Total Response Interval. Note that the red arrow exemplifies a negative lag [or overlap], while the yellow arrow exemplifies a positive lag [or alternation].

The results depend upon our definitions of the notions response and response interval, which must be made explicit in order for our effort to be replicable. A Total Response Interval was defined to begin at 0.2 s after the onset of a referent utterance (by the mother or the baby) and to end at 3 s after the offset of the referent utterance (see Figure 1, upper green arrow). In order for an utterance to be deemed a response, it had to *start* within the Total Response Interval. The two purple boxes in Figure 1, are not deemed to be responses because their start times do not fall within the Total Response Interval. When defining the responses and calculating lag times, we apply three criteria with regard to the onset and offset of referent and responsive vocalizations. First, as indicated in stippled blue, in order to be treated as a response, a vocalization (e.g., *Vocal response 1* in orange) must begin > 0.2 s after the onset of the referent vocalization (the entire vocalization includes both stippled blue and solid blue). Thus, the first purple vocalization does not qualify as a response because it does not begin after the first 0.2 s of the referent vocalization. *Vocal response 1* and *Vocal response 2*, however, both qualify. Second, a response that follows the offset of the referent must begin within 3 s (bottom green arrow) of that offset. Thus, the second purple vocalization does not qualify as a response because it begins > 3 s after the offset of the referent vocalization. Third, there is a one-to-one mapping of any referent vocalization to a response, and the response must be the first vocalization to qualify. Thus, in Figure 1, *Vocal response 2* could only count in the analysis if *Vocal response 1* had not occurred. Any vocalization that qualifies as responsive to more than one referent, must be assigned, in accord with the one-to-one mapping rule, to just one of those potential referents, namely the one whose onset is closest in time to the onset of the potential response. Finally, note that the total response interval duration (upper green arrow) for any referent vocalization, can vary substantially, because it depends on the duration of the referent vocalization itself plus the 3 s following interval.

**FIGURE 2:**
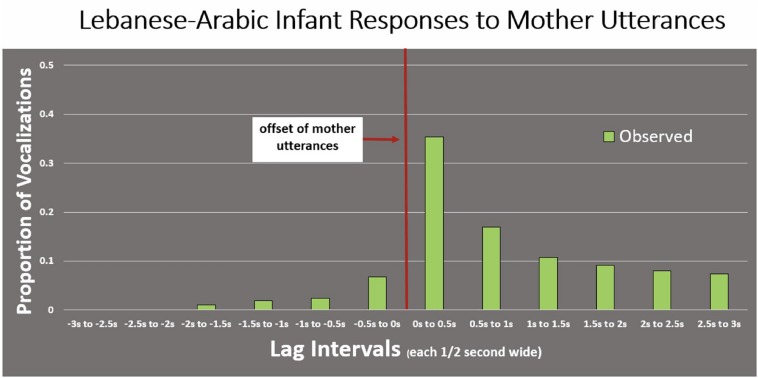
Observed data for proportion of infant vocal responses to mother utterances averaged across the Lebanese-Arabic dyads. The display shows that the great majority of infant utterances followed the offset of mother utterances, suggesting conversational alternation and coordination. This display also shows that the infant responses occurred most frequently right after the mother utterances ended, in the first 0.5 s after the offset of maternal utterances. However, comparison with a randomized distribution is required in order to assess infant vocal responsivity more comprehensively (see subsequent sections).

The audio displays allowed ms-level resolution for the visualization of onsets and offsets of utterances. The 0.2 and 3 s time frames are empirically motivated. A vocal response to an auditory stimulus requires about the duration of a short syllable (∼ 0.2 s, Jain et al., 2015), and consequently a vocalization occurring in the 0.2 s interval after the onset of another vocalization should not be considered a response. Further, a pause within an adult conversation of more than 3–5 s can often be seen to correspond to a topic change; an utterance occurring after such an interval can reasonably be thought of as a new initiation of the old conversation or the beginning for a brand new one. The 3 s time frame seemed empirically appropriate because, for the data displayed below (illustrated in Figure 2), we found that the high responsivity shortly after the offset of referent utterances decreased rapidly thereafter, and if we extended the distributions beyond 3 s, the proportions of “responsive utterances” ceased to decrease further, suggesting that after 3 s, speakers were randomly initiating potentially new conversations, rather than responding to a prior utterance of the other speaker. Thus empirically the 3 s frame after the end of a referent utterance seemed to encompass the real responses to that referent utterance. Figure 1 illustrates our definitions.

Next we illustrate the response interval idea based on the actual distribution of the Lebanese-Arabic observed data for infants responding to mothers (Figure 2). This distribution shows the observed data averaged across the Lebanese-Arabic dyads, where the proportions of infant utterances that met the requirements to be responses to mother utterances within each time interval are displayed. Thus, the first bar to the right of the vertical red line indicates the proportion of infant vocal responses occurring in the first half-second after the offset of the mother utterance, while the next bar to the right represents the infant responses occurring in the second half-second. Bars to the right of the red line correspond to *positive lag*, that is proportions of infant utterances that followed the offset of mother utterances. Bars on the left represent the proportion with *negative lag*, that is infant utterances that *preceded* the offset of mother utterances, and thus *overlapped* with them. Clearly, the great majority of infant utterances followed the offset of the mother utterances, suggesting conversational alternation and coordination. It is clear that infant responses occurred most frequently right after the mother utterances ended, in the first half-second after the mother offset. At the same time, while Figure 2 suggests that the Arabic infants were alternating with their mothers vocally, the pattern could be misleading unless we compare the observed distributions with randomly distributed possible infant responses, which we address next.

### Generating Randomized Distributions and Testing for Responsivity

We conducted Kolmogorov–Smirnov (K–S) tests to examine whether the observed responsivity patterns differed from randomly generated simulations of responsivity patterns in both Arabic and English for mothers responding to infants and for infants responding to mothers. The following describes the generation of the randomized distributions, which was based on principles in common usage in the literature on randomized designs (Kempthorne, 1955; Edgington, 1969; Romano, 1990; Gadbury, 2001). This kind of randomized approach has been widely specified and used in a variety of fields, although to our knowledge has not been used before in parent-infant interaction research. Our intent is to specify our procedure sufficiently for potential replication. Supplementary Appendices A and B provide both a technical description of the procedure and the R code that implemented it. In the following we describe the procedure conceptually.

A “pair” of utterances was defined here as consisting of a sequence of a mother utterance (the referent) followed by an infant utterance (the response), or an infant utterance (the referent) followed by a mother utterance (the response), meeting the timing requirements of our response interval definition as in Figure 1. Thus, a response always started at least 0.2 s after the onset of the referent. Further, a response could start up to 3 s after the offset of the referent.

For each of the four data sets (Arabic mothers responding to Arabic infants, American mothers responding to American infants, Arabic infants responding to Arabic mothers, American infants responding to American mothers), the mean duration of the responses and the mean duration of the pauses between the responses was computed for each recording. Using these means as parameters of exponential distributions, a sequence of random response durations and pause durations was generated for each recording. This randomized sequence of (possible) responses was compared with the fixed set of referents within each recording to calculate the lags (either positive or negative) corresponding to the qualifying responses (in accord with the response definition) of the randomized distribution for that recording. One thousand such random sets were generated for each recording, and the corresponding lags were computed for each set of infant-mother or mother-infant pairs across each set of recordings from each of the four data sets. Randomized distributions for each of the four data sets represented data collapsed across all the recordings within each data set.

**FIGURE 3:**
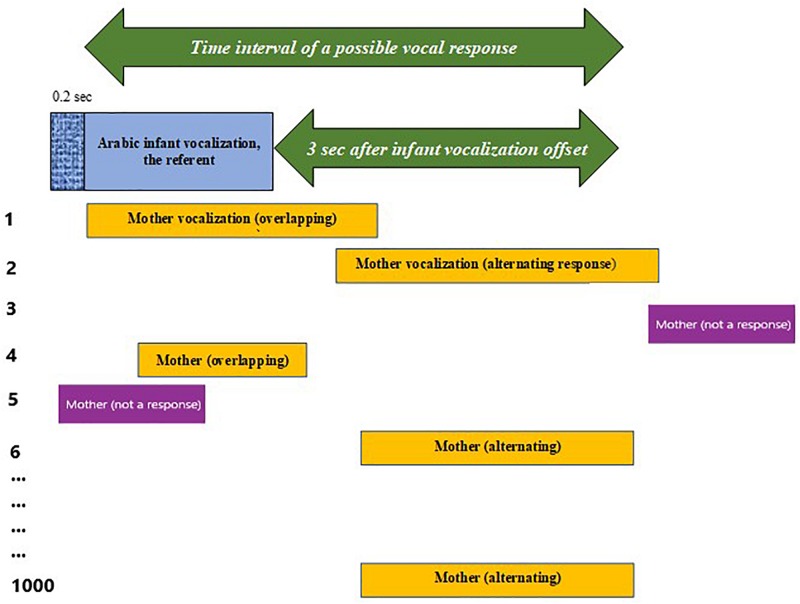
An illustration of how simulated responses were generated for the randomized distributions. A single infant utterance with the time interval of a possible response specified (green arrow), along with 6 randomly generated potential mother responses (out of 1000) selected from the distribution of the mean durations of the observed mother utterances and spaced so that the pause durations were also drawn from the distribution of the mean of the pause durations between observed mother utterances in the recording. The lags of randomly generated mother vocalizations qualifying as responses were calculated for the 1000 sets. The Kolmogorov–Smirnov (K–S) test compared the outcome of the randomized distributions with that of the observed distributions 1000 times.

For example, in a K–S test for mother responses to infant utterances in Arabic, we started by fixing in time the infant utterances (in this case, the referents) for an Arabic recording. Figure 3 shows an example infant utterance placed in time as it was observed, with the time interval of a possible response specified. Also displayed are six possible randomly generated potential mother responses (out of the 1000 generated for each recording), the durations of which were all selected from the distribution of the mean durations of the observed mother utterances in the Arabic recording in question and all spaced within each of the set of 1000 so that the pause durations were also drawn from the distribution of the mean of the pause durations between mother utterances. Having generated the randomized sets, the lags of randomly generated mother vocalizations qualifying as responses were calculated for the 1000 sets, and the outcome responsivity for the randomized distributions was compared with that of the observed distributions 1000 times by the K–S test.

## Results

### Illustration of Comparisons Between Observed and Randomized Distributions of Vocal Responses: The Case of Lebanese Arabic-Learning Infants Vocally Responding to Their Mothers’ Vocalizations

Understanding the results requires appreciation of an asymmetry in the shapes of the randomized distributions obtained through our procedure. One might expect random events to be distributed roughly equivalently across the time frame evaluated for responses. However, actually randomized events show much larger numbers of positive than negative lag values, and are thus more similar to the observed distributions of responses than one might imagine. This asymmetry in the randomized distributions is the result of asymmetry in the durations of referent utterances and the following response intervals. The former were variable (typically from 0.4 to 1 s for infant utterances, and 0.5 to 1.5 s for mother utterances) and much shorter than the fixed following response intervals (3 s).

**FIGURE 4:**
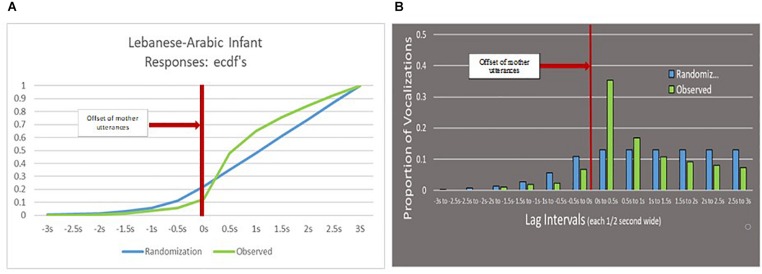
**(A)** In the Left panel, showing empirical cumulative distribution functions (ecdfs), the blue line represents the results of the randomized simulation of infant responses to the mother utterances as empirical cumulative distribution functions (which rise from 0 to 1 left to right). The line begins to rise clearly at –1.5 s and then rises linearly from 0 to 3 s. The green line represents the real infant responses, which remain lower than the randomized responses prior to the 0 point, but abruptly exceed the randomized responses shortly after the 0 point. The significant K–S test, *p* < 0.001, suggests Arabic infants responded more than expected by chance shortly after the mother utterance offset and less than expected by chance shortly before the mother offset. **(B)** In the Right panel, the blue bars represent the same results as in **(A)**, but in this case in terms of *proportions* of observed and randomized responses in each interval. Thus, in both **(A,B)**, the real tendency of the infant to alternate conversationally is reflected in the *differences* between the randomized and observed data.

We conducted the K–S test to examine whether our observed data (either mother or infant vocal responses) showed random or systematic responses with respect to the referent utterances. If the distribution of the observed data significantly differs from the distribution of the randomized data, it corroborates that the observed data indeed show systematic behaviors. Figures 4A,B provide examples of the K–S test and we provide details of the analysis as follows.

Figure 4A (empirical cumulative distribution functions of responses, ecdfs) and Figure 4B (proportional occurrence of responses) illustrate the outcome for Lebanese infants responding to Lebanese mothers in two ways. In Figure 4A the outcome is displayed as two empirical cumulative distribution functions (ecdfs) for the observed and randomized lags, while in Figure 4B the same data are displayed in bar-graph form, with each bar representing proportions of responses in the observed and randomized data (in this illustration, the observed data are the same as in Figure 2). In both cases, the data are binned at 0.5 s starting 3 s before the 0 point (the offset of the referent utterance, by the mother in this case) to 3 s after the 0 point. Because the referent utterances were of variable duration, and were typically much shorter than 3 s, there are very few responses in the Figures for either the observed or the randomized distributions prior to a lag of −1.5 s.

The slow rise in the negative lag range for both Figures 4A,B and the much more rapid rise in the positive lag range are of course attributable to the much lower likelihood of a responsive utterance occurring *during* a mother referent utterance (which was typically much less than 3 s in duration) than during the following pause (which was typically three or more times longer). In Figure 4A the randomized distribution rises essentially linearly in the positive lag range, and in Figure 4B the same distribution is essentially flat in the positive lag range, because the positive lag range is fixed in duration. It is the relative positioning of the observed and randomized data in these figures that supply the most meaningful portrayal of infant vocal responsivity—in the absence of the randomized comparison, the responsivity patterns of the infants in Figures 4A,B would appear misleadingly large, because we would be tempted to base our judgment only on the relative amounts of response before and after the 0 point.

The K–S test for whether the observed Lebanese Arabic-learning infant responses were random with respect to the fixed Lebanese Arabic-speaking mother speech patterns rejected the null hypothesis at *p* < 0.001 (test statistic = 0.168), indicating that Lebanese infants did indeed respond systematically. In Figure 4A, it can be seen that the two distributions begin to separate in the 1.5 s before the offset of the referent mother utterance (the 0 point) indicating the tendency of the real infants (in green) to respond less often with negative lag or overlap than in the case of randomly distributed simulated responses (in blue). Then immediately after the offset of the mother referent utterance, the real infant responses abruptly rise above the randomized responses, indicating infants tended to respond non-randomly, especially in the first 0.5 s after the mother utterance offset. In Figure 4B, the observed infant responses also show lower proportions prior to the mother utterance offset than randomly occurring simulated responses, with a sharply higher proportion for the observed infant responses in the 0.5 s after the mother utterance offset. Clearly, as seen in Figure 4B, the Lebanese infants seemed to cluster their responses shortly after the offset of the mother utterances, suggesting a pattern of conversational alternation.

### Summary of Various Comparisons of Observed and Randomized Distributions of Vocal Responses

The relation between response patterns as seen in the ecdfs and in the proportion displays is similar for all the comparisons to follow, and K–S tests produced significant rejection of the null hypothesis that the observed and randomized distributions were the same at *p* < 0.001 for all the four key cases discussed below (Lebanese infants responding to Lebanese mothers [as indicated above], Lebanese mothers responding to Lebanese infants, American infants responding to American mothers, and American mothers responding to American infants). Moreover, the data show a similar pattern in all four cases, in that the responders showed fewer vocalization responses in the half-second interval before the referent speaker’s offset than in the randomized set, and more responses in the half-second interval after the referent speaker’s offset than in the randomized set.

Although not the focus of the study, we conducted exploratory analyses to examine whether the temporal structure of mother-child vocalizations varied as a function of children’s age. The infants followed the same pattern of vocal responsivity at both the younger (<10 mo) and older ages (>10 mo) in both cultural groups with all four comparisons of observed and randomized distributions, in all cases showing *p* < 0.001. As expected, mothers showed more distinct responsivity than infants in both cultural groups (*p* < 0.001), with higher proportions of responses in the first half-second after the infant referent vocalizations than the infants showed with respect to the mother referent vocalizations.

For the remaining displays in the main text of the article, we shall present the data in terms of observed and randomized *proportions* only. Additional ecdfs are provided in Supplementary Appendix C. It might be speculated that with our relatively small N, outlier infants might have strongly affected the patterns of results. We have supplied ecdfs in Supplementary Appendix C that show observed lag distributions for the individual infants, revealing that outliers played a small role, with most infants conforming to the patterns reported below.

### Arabic-Learning Infants Compared to Arabic-Speaking Mothers

**FIGURE 5:**
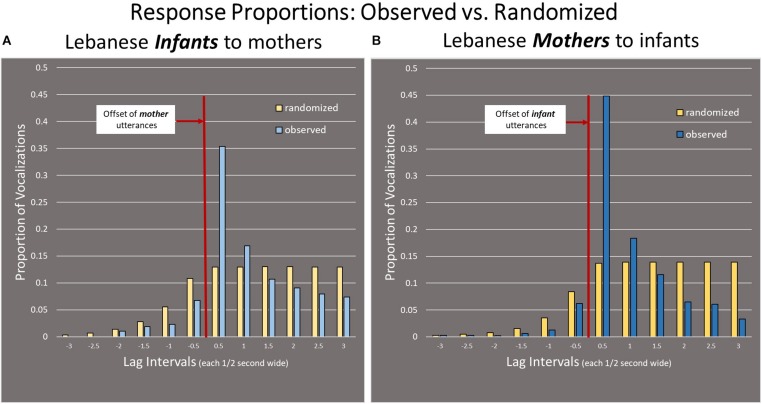
Comparison of infant and mother responses in the Lebanese group. **(A)** The Left panel, shows that Arabic-learning Lebanese infants were vocally taking turns with their mothers. **(B)** The Right panel shows that Arabic-speaking Lebanese mothers were also vocally alternating with their infants. For both infants’ and mothers’ responses, the greatest proportion of responses occurred during the first 0.5 s interval after the offset of the utterances. Mothers responded quickly to infants more consistently than infants responded quickly to mothers, as indicated by a higher proportion of mother responses in the first half-second after the 0 point.

In Figure 5, the Arabic-learning *infants* as responders are shown on the Left panel and the Arabic-speaking *mothers* as responders are shown on the Right panel. Here again, the intervals on the x-axis represent half-second time frames, from −3 s to +3 s with respect to offset of the referent utterances. The results indicate that the mothers alternated vocalizations with infants in a similar way to that of the infants alternating with the mothers. The greatest proportion of responses of mothers also occurred during the first half-second interval after offset of the infant utterances, and there was a tendency for fewer responses to occur in the intervals before the infant offset than in the case of randomly distributed responses. Moreover, the mothers were more consistent in responding quickly than the infants were (*p* < 0.001)—45% of responses of mothers versus 35% of responses of infants were in the first half-second after the offset of the referent utterance, showing that Lebanese mothers were more precisely able to time their vocally responsive utterances than infants.

### English-Learning American Infants Compared to English-Speaking American Mothers

**FIGURE 6:**
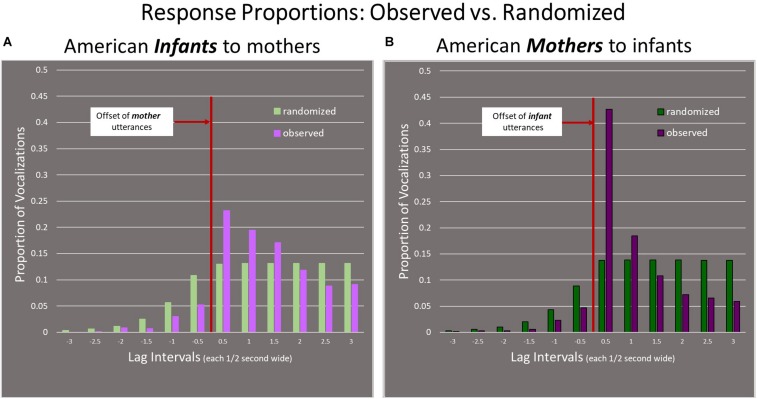
Comparison of infant and mother responses in the American group. **(A)** The Left panel shows that English-learning American infants were vocally taking turns with their mothers. **(B)** The Right panel shows that English-speaking American mothers were also vocally alternating with their infants. The greatest proportion of responses occurred during the first half-second after the offset of the utterances. The English-learning infants’ preference for responding in the first half-second was not as strong as in the case of the English-speaking mothers.

Figure 6 illustrates the data for the American dyads with infants on the Left panel and mothers on the Right panel. As in the Lebanese Arabic case, the K–S test showed there was a statistically significant difference from chance for both infants and mothers (*p* < 0.001). Similar to the Lebanese Arabic data, the greatest proportion of responses of infants and mothers occurred during the first half-second after the referent utterance offset. It is thus evident that both mothers and infants in both cultures showed significant vocal alternation. As in the case of the Lebanese dyads, the American infants’ preference for responding in the first half-second was much less strong than in the case of the American mothers (*p* < 0.001), with 42% of maternal responses occurring in the first half-second compared to only 23% of infant responses.

### American Infants Compared to Lebanese Infants

**FIGURE 7:**
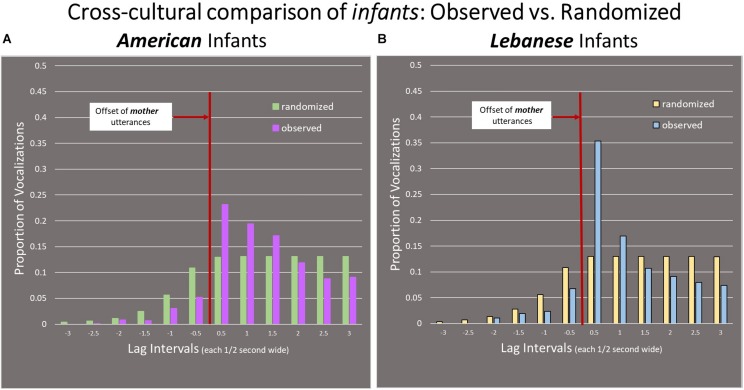
Comparison of infant vocal responses in both cultural groups. **(A)** Arabic-learning Lebanese infants (Right panel) tended to respond more heavily in the first 0.5 s interval compared to **(B)** the English-learning American infants (Left panel).

Based on data in Figure 7, we conducted the K–S statistical significance test for possible differences between American and Lebanese Arabic infants and found that Lebanese Arabic infants tended to respond more heavily in the first half-second interval after mother utterance offset compared to the American infants (the lag distributions of observed responses differed across cultural groups at *p* < 0.001; the K–S test statistic was 0.14 with the critical value for *p* < 0.001 being 0.09). The pattern shows 35% of Arabic infants responses occurred in the first half-second compared to 23% for the American infants. In fact, the American infants continued to show response levels exceeding chance even in the third interval, out to a second and a half beyond the offset of the mother utterances. In contrast, the Lebanese Arabic infants’ responsivity beyond the chance level did not continue beyond the one second period after the offset of the mother utterances.

### American Mothers Compared to Lebanese Mothers

**FIGURE 8:**
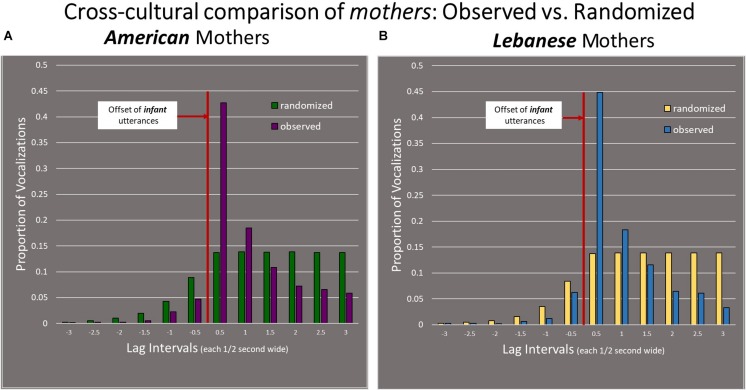
Comparison of mother vocal responses in both cultural groups. **(A)** American mothers’ responses and **(B)** Lebanese mothers’ responses. Unlike the infants, the pattern of mothers’ responses to infants did not differ across cultures/languages.

The mothers did not differ in the way the infants did across cultures (Figure 8). In both cultures, the responses of mothers were concentrated within the first half-second after the offset of the infant utterances, and the proportions of responses within that interval were similar (42 and 45%, *p* > 0.05; the test statistic was 0.03808, while the critical value for *p* < 0.05 was 0.0624).

## Discussion

The current study sought to investigate temporal coordination in vocal exchanges between mothers and infants across two cultural groups, Lebanese-Arabic and American-English dyads in the first 2 years of life. Our method addressed both gaps and overlaps in both mother–infant and infant–mother responses. The study aimed at answering three research questions:

(1) What is the timing of mothers’ vocalizations in response to their infants’ vocalizations within and across cultures?

We found that the mothers appeared to engage in vocal alternation and behave similarly in terms of vocal response timing with their infants within and across Lebanese and American cultures and across the first 2 years of life. This suggests that mothers may be responsive to their infants’ vocalizations in both cultures and may perceive their infants as capable communication partners, thus treating their vocalizations as intentional communication bids (Schieffelin and Ochs, 1986).

(2) What is the timing of infants’ vocalizations in response to their mothers’ vocalizations within and across cultures?

Similarly, our findings reveal within each culture that infants seem to respond contingently to mothers’ vocalizations: Lebanese infants followed Lebanese mothers vocally, and American infants followed American mothers vocally. These patterns support the view that temporal interactional dynamics is a universal phenomenon (Stivers et al., 2009). That this pattern applied both to the Lebanese and American dyads and to infants across the first and the second year suggests that contingent vocal interaction with alternation is a fundamental characteristic of humanity, supporting the supposition that such interaction forms a foundation for language (Trevarthen, 1979, 2001).

While both mothers and infants showed systematic patterns of vocal response within the first half-second after the offset of vocalizations by the other speaker, the tendency to favor that first half-second was notably stronger in the mothers than the infants in both cultures. The pattern suggests that mothers across the two cultures may have been guiding their infants, whether consciously or unconsciously, toward conversational competence. Other recent studies suggest that mothers, even in the first month, engage in such conversational guidance (Dominguez et al., 2016; Yoo et al., 2018). In fact, it is not entirely clear in the first few months of age that infants actively respond to vocalizations of mothers at all. We cannot rule out the possibility that mothers are able to predict infant vocal patterns and that they tend to curtail their own vocalizations when they sense their babies are about to speak. If this is true, their predictive curtailments would surely yield the impression that their infants are more actively involved in conversation than they actually are.

By 5–6 months of age, there exists other evidence that infants do indeed actively play a role in conversation—they tend to double their rate of vocalization in the still-face paradigm during the still-face episode, indicating presumably that they are trying to repair the “broken” conversation when the mother goes silent (Yale et al., 2003; Goldstein et al., 2009; Franklin et al., 2013).

These thoughts are important in the present context because they highlight the fact that even our own pattern of results—comparing observed and randomized distributions—does not rule out a role for the mother in guiding conversations with her infant, even at the older ages of the study, producing patterns that may suggest a more systematic and active role for the infant than is veridical. Surely, infants are learning to predict mothers’ vocal patterns across the first years of life, and they might also be learning to guide conversations, as the mothers clearly do. It is important to note that while our method shows unambiguously that there are systematic relations in time between mother and infant vocalizations, and that those timing relations suggest conversation, the results cannot show by themselves that infants are responding actively, given that mothers clearly have at least some ability to predict infant vocalization timing. We do, however, believe it is safe to conclude that the results suggest active entrainment by the mothers, encouraging infants to learn the patterns of contingency that conversation requires.

(3) Are the patterns of conversational turns different across cultures?

Despite the observed universal pattern of vocal responsivity in mothers and infants, some cultural variation emerged. While mothers responded to infant vocalizations in a similar time frame across the two cultures, we found cross-cultural differences in the infants’ responsivity. These differences were observed in the speed with which Lebanese infants responded to their mothers, with more than one third of the Lebanese infant responsive utterances tending to occur within a half-second after the offset of maternal utterances, compared to less than one quarter in the American infant sample.

While the mechanisms that might drive infants to show different patterns of temporal responses across cultures are unknown, here we provide our favored speculation, which is based on the possibility that the Arabic language may provide more predictable indicators of utterance termination than the English language. We are not asserting this difference to exist—both languages have complex stress systems (Kenstowicz and Abdul-Karim, 1980; McCarthy, 1980; Selkirk, 1980; Osterling and Dawson, 1994; Kiparsky, 2003; Watson, 2011)— but if it does, it could help explain the infant responsivity pattern, where Lebanese Arabic infants seemed more able than American English infants to time their responses with the ending of mother utterances.

Let us exemplify: We speculate that English may not be as predictable in terms of location of stress in words as Arabic (Watson, 2011). Word stress is well known in some languages to be a strong predictor of boundaries. For example, French tends to have final word stress, Czech initial stress, and both Polish and Spanish penultimate. Thus, any content word pronounced in any of these languages provides a clue about when it may end as soon as the stress is identified by the listener in real time. Both English and Arabic have very complicated stress patterns. In fact, recent evidence suggests that stress is highly predictable in Modern standard Arabic (Saiegh-Haddad and Henkin-Roitfarb, 2014) and the Cairene (Egyptian) dialect of Arabic (Aquil, 2011). Accordingly, our suggestion is that perhaps the English stress is the less predictable and consequently, the American infants may have tended to lag a little longer in their responses, because they did not have such a strong basis as the Lebanese infants to predict when the mothers’ utterances would end.

An alternative interpretation for the differences in infant temporal coordination across the cultures could be based on differences in maternal utterance duration (Farran et al., 2016). IDS has been thought to encourage rapid infant vocal responsivity, and short IDS utterances may be particularly effective in facilitating rapid responsivity. It could be the case that the Lebanese infants’ ability to respond quicker to their mothers’ utterances was brought about by Lebanese mothers’ shorter utterance duration (and Lebanese infants’ awareness of it) compared to that of American mothers. Similarly, intonation patterns could have differed between the languages, cuing the Lebanese infants more fully than American infants about maternal utterance termination.

Our aim at offering a culturally motivated explanation leads us to a related speculation. The fact that infants across the two cultural groups differed in their ability to time their turns to match their mothers’ communicative bids could be driven by the differing mothers’ expectations of their children, owing to potential cross-cultural differences in child rearing practices and the degree to which infants are conceptualized as communicative beings (Goodnow et al., 1984; Keller et al., 2007). The Lebanese mothers may have thus been more likely to converse using significantly shorter, simpler utterances, which in turn facilitated infants’ ability to co-coordinate the timing of their vocalizations to match that of their mothers. At the same time, it is important to emphasize that mothers in both cultural groups appeared to be seamlessly and effortlessly capable of tailoring the timing of their vocalizations to adjust to the timing of their infants’ vocalizations, presumably enabling both groups of mothers to recruit their infants’ rudimentary turn-taking abilities toward increasingly sophisticated language competence (Fernald et al., 1989).

The fact that both mothers’ and infants’ vocalizations were temporally structured, corroborates previous research (Jaffe et al., 2001; Keller et al., 2007; Bornstein et al., 2015) pointing to sensitivity on the part of the dyad to adapt to the cultural environment (for example to patterns of interaction of the other party to conversation or to special characteristics of the language structure) (Thelen and Smith, 1994). The results suggest it may be important to consider not only universal variables in child rearing practices, but also cultural and language-specific variables which may shape social interaction uniquely, thus creating special cultural niches (Odling-Smee, 1988) between mothers and their infants that form the basis for the development of language in infancy and early childhood.

Several efforts have aimed at gaining a fuller understanding of vocal development across cultures (Bornstein et al., 2015) and socioeconomic strata (Hoff, 2003). While the evidence to date corroborates IDS and vocal coordination between infants and their mothers across a variety of cultures, it has also been speculated that vocal coordination may be derailed, if, for example, endogenous infant vocalization or maternal response to infant vocalizations is very low (Oller et al., 2019). The current study supports the need to investigate language structure and cultural practices as important influences on of vocal coordination.

Understanding how vocal interaction unfolds between typically developing infants and their mothers also has the potential to inform our understanding of the emergence of developmental anomalies (Leezenbaum et al., 2014) across cultures. This may in turn be crucial in contributing to the assessment of and intervention with infants and young children who have developmental vulnerabilities (e.g., ASD, see Northrup and Iverson, 2015) and shaping developmental trajectories of turn-taking for optimal language outcomes across cultures.

Future research attention should be directed to understanding the genesis of turn-taking between mothers and their infants with developmental disorders (e.g., ASD) across cultures and languages, characterizing the contribution of each member of the dyad in the co-coordination of communicative exchanges. Moreover, future studies could help uncover the mechanisms that undergird maternal input and interactivity such as varying arousal levels of infants associated with different speech registers (Farran et al., 2016), level of infant volubility (Oller et al., 2019) and lexical diversity, and varying cultural views of infants thought to be associated with the emergence of language in human infancy (Kärtner et al., 2010). A challenge for future work will be to tease apart the input cues (e.g., acoustic, visual, physical) that likely interact with infant characteristics (e.g., presence of a developmental disorder, communicative fitness, attachment) to shape the trajectories of turn-taking in infants at-risk for or diagnosed with developmental disorders (Loveland, 1991). Current research points to the importance of multimodal sampling of vocal interaction—a limitation of the present study. This is particularly crucial given evidence underscoring the importance of the visual modality in infant volubility (blind infants have been reported to show low volubility, Fraiberg, 1979) and the potential protective role multimodal cues may exert in cases where maternal input has been found to be less-than-optimal (e.g., low SES, low technology societies) (Cristia et al., 2017). Such an understanding will contribute immensely to the design and implementation of better intervention studies that may ultimately improve future language and overall developmental outcomes of at-risk children. Future research could also benefit from employing more nuanced approaches to data collection and analysis by introducing random sampling of home recordings of face-to-face interactions, multimodal data collection, and targeting languages that come from different language families as well as various dialects within specific languages such as Arabic and English. Such expanded research should enable direct evaluation of the contribution of various language and dialect structures to early vocal coordination and ultimately language emergence across cultures.

## Data Availability Statement

The datasets generated for this study will not be made publicly available. The recordings cannot be made publicly available because the conditions of IRB approval for making the recordings do not include permission from the parents to make the recordings available publicly. To provide the raw recordings would violate the confidentiality requirements of the data collection. However, we can supply the data sheets including durations obtained from both coders. From these sheets, all the calculations have been made that are included in the paper. Requests to access the datasets should be directed to the corresponding author.

## Ethics Statement

The University of West Georgia Institutional Review Board and the University of Memphis Institutional Review Board approved this study. This study was carried out in accordance with the recommendations of the University of West Georgia and University of Memphis guidelines and IRB Committees’ approval with written informed consent from all subjects. All subjects gave written informed consent in accordance with the Declaration of Helsinki. The protocol was approved by the IRB committee at the University off West Georgia and the University of Memphis.

## Author Contributions

LF and DO contributed to conception and design of the study. LF, HY, and C-CL organized the database. DB performed the statistical analysis. LF wrote the first draft of the manuscript. LF, DB, and DO wrote sections of the manuscript. All authors contributed to manuscript revision, read and approved the submitted version.

## Conflict of Interest

The authors declare that the research was conducted in the absence of any commercial or financial relationships that could be construed as a potential conflict of interest.
