# Supplementary material for: Temporal Coordination in Mother–Infant Vocal Interaction: A Cross-Cultural Comparison
Source: Front Psychol. 2019 Nov 8;10:2374. doi: 10.3389/fpsyg.2019.02374 (PMC6856762; doi:10.3389/fpsyg.2019.02374)
Supplement: Supplementary file 1 [file Table_1.docx]

**Appendix A: Technical description of randomization distribution plan for permutation test**

# Introduction

For a single caregiver/infant conversation let $X_{ib}$ be the time at which the *i*th caregiver utterance begins and $X_{ie}$ be the time at which the *i*th caregiver utterance ends. Then $X_{ie}-X_{ib}$ is the duration of the *i*th utterance. Here $i=1,\ldots,n,$where *n* is the number of caregiver utterances in $(0,T)$ and $T$ is the time at the end of the session.

Over the same time interval, let $Y_{ib}$ be the time at which the *i*th infant protophone begins and $Y_{ie}$ be the time at which the *i*th infant protophone ends. Similarly, $Y_{ie}-Y_{ib}$ is the duration of the *i*th infant utterance. Here $i=1, \ldots, m$, the number of infant utterances in $(0,T)$.

Define the caregiver interval as ${(X}_{ib}, X_{ie}+3000ms)$ for $i=1, \ldots, n$. Then if there exists $Y_{jb}\in(X_{ib}, X_{ie}+3000)$, define the observed infant lag as

$$\mathcal{l}_{k}=Y_{jb}-X_{ie}.$$

The distribution of $\mathcal{l}_{k}$’s is of interest. A histogram (or estimated smooth density) of the observed infant lags is created using all caregiver/infant conversations. We will compare the observed distribution of infant lags with a random distribution of infant lags.

# Randomization Distribution

Again for a single caregiver/infant conversation consider every caregiver interval, $(X_{ib}, X_{ie}+3000)$, $i=1, \ldots, n.$ Generate $U_{1}, \ldots, U_{n}$ where $U_{i}$ is uniformly distributed on $(X_{ib}, X_{ie}+3000)$ and calculate the generated lags as $L_{i}=U_{i}-X_{ie}$. Repeat this $N$ times to generate a distribution of uniform lags $L_{i}$’s. Use the generated lags of all caregiver/infant conversations to obtain a histogram (or estimated smooth density). Compare the uniform lag distribution to the observed lag distribution. A qqplot can be used to visually compare the distributions and a non-parametric test such as the Kolmogorov Smirnov test can be used to test whether the distributions are the same. A permutation test can be performed to test whether medians/means of the distributions are equal as well.

# Randomization Including Duration

For a single caregiver/infant conversation, fix the caregiver’s conversation, $\{\left( X_{ib},X_{ie} \right), i=1, \ldots, n\}$. We want to generate random infant streams to overlay on this set. For the observed infant conversation define

$$T_{i}=Y_{ie}-Y_{ib}$$

as the duration of the infant protophone $i$, for $i=1, \ldots, m$ and

$$V_{i}=Y_{ib}-Y_{i-1,e}.$$

For $Y_{0e}=0$ and $Y_{m+1,b}=T$, the $V_{i}$’s are the inter-arrival times, $i=1, \ldots, m+1$. Two schemes are proposed for generating the random infant streams.

## Random Scheme 1

Assume the process $\left\{ Y_{1b}, Y_{1e}, \ldots,Y_{mb}, Y_{me} \right\}$ is a Poisson process with mean number of events $\lambda T.$ Estimate $\lambda$ by $\frac{2m}{T}$. Then generate the random infant stream as $\left\{ 0, U_{1b}, U_{1e}, \ldots, U_{me}, T \right\}$ by generating the interarrival times $W_{i}$ between each event. Thus $W_{1}=U_{1b}-0, W_{2}=U_{1e}-U_{1b}$, etc. Since we have assumed a Poisson process for the events, the interarrival times will be independent exponential random variables with mean $1/\lambda$, i.e. the pdf is given by

$$f\left( w \right)=\lambda e^{-\lambda w}.$$

The estimated lambda for each caregiver/infant conversation can be used to randomly generate the infant streams.

## Random Scheme 2

In this scheme we assume the distributions of durations, $T_{i}$’s and inter-arrival times, $V_{i}$’s are different, independent exponentials. Suppose $T_{i}\sim exponential(\lambda)$ and $V_{i}\sim exponential(\theta)$. Here $\lambda$ can be estimated as the mean duration,

$$\bar{T}= \frac{1}{m}\sum_{i=1}^{m} T_{i}$$

and $\theta$ can be estimated as the mean inter-arrival time,

$$\bar{V}=\frac{1}{m+1}\sum_{i=1}^{m+1} V_{i.}$$

In this case the $T_{i}$’s are generated from

$$f\left( t \right)=\frac{1}{\lambda}e^{-t/\lambda}$$

and the $V_{i}$’s are generated from

$$f\left( v \right)=\frac{1}{\theta}e^{-v/\theta}.$$

Then the random infant streams can be found as

$$\left( 0, U_{1b} \right) \left( U_{1b}, U_{1e} \right) \ldots\left( U_{mb}, U_{me} \right) (U_{me}, T)$$

$$V_{1} T_{1} \ldots T_{m} V_{m+1}$$

where $U_{1b}=V_{1}, U_{1e}=U_{1b}+T_{1},$ etc.

## Computing Lags

For either scheme for random infant streams, using $U_{1b}, U_{1e}, \ldots, U_{mb}, U_{me}$ as the infant stream, compute the infant lags as follows: If there exists a $U_{jb}\in(X_{ib}, X_{ie}+3000)$ compute the lags as $L_{k}=U_{jb}-X_{ie}$ for $i=1, \ldots, n$. This randomized distribution of lags can be compared to the observed lags using the methods previously discussed.

**Appendix B: R code for randomization distribution**

###data is input as two columns indicating the beginning and ending

##utterance times.

data<-read.csv("arabicparent.csv",header=F)

###get beginning times Xb and ending times Xe

Xb<-data$V1

Xe<-data$V2

n<-length(Xb)

lag<-NULL

k<-1

##m is the number of utterances in the time interval

m<-184

###st is starting time

st<-560793

###lambda is the mean duration time

lambda<-892

###theta is the mean interarrival time

theta<-2371

## generate random distribution

for (l in (1:1000)){

##Generate T's and V's to get the randomly generated Intervals

v<--theta*log(1-runif(m))

t<--lambda*log(1-runif(m))

###compute the beginning and ending times for the generated T and V

Ub<-NULL

Ue<-NULL

Ub[1]<-st+v[1]

Ue[1]<-Ub[1]+t[1]

for (i in 2:m){

Ub[i]<-Ue[i-1] + v[i]

Ue[i]<-Ub[i]+t[i]

}

###Calculate the lags

for (j in 1:m){

for (i in 1:n){

if (Ub[j]>=Xb[i] & Ub[j]<=Xe[i]) {

lag[k]<-Ub[j]-Xe[i]+3000

k<-k+1

# print(i)

}

}

}

}

lag<-lag/100

##output a set of random lags

write.csv(lag,"lagbaby1.csv")

**Appendix C: Empirical Cumulative Distribution Functions**

| **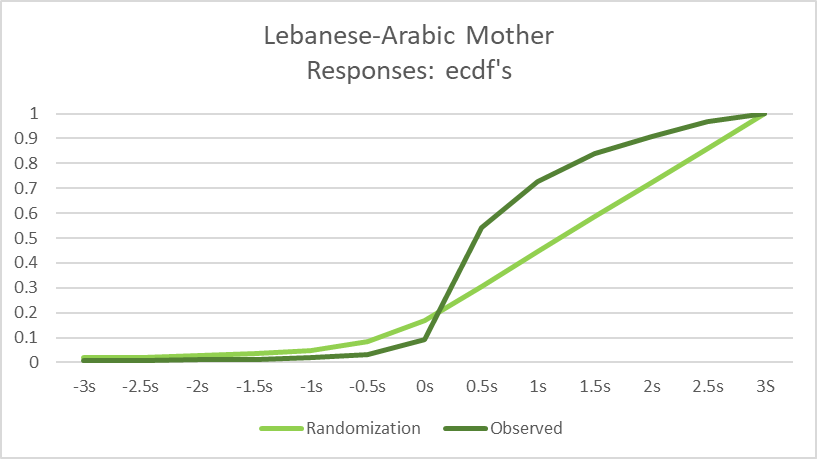** | **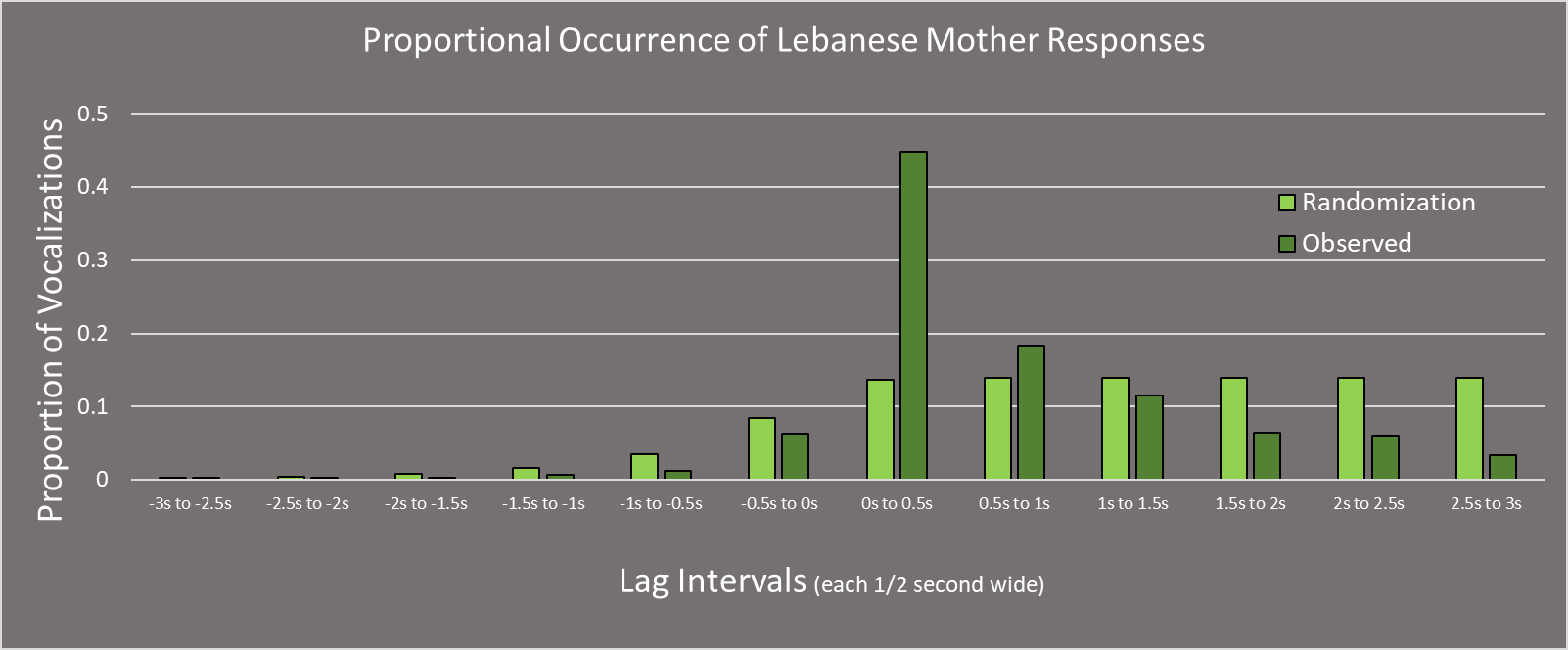** |
| --- | --- |
| **Figure C1** | |

**Figure C1: Arabic Mothers Randomized Distribution**

The K-S test rejected the null hypothesis that the observed lags and the randomized lags for the Lebanese Arabic parents came from the same distribution with p-value < 0.001 (test statistic = 0.28). Figure C1 shows the empirical cumulative distribution functions (ecdf) for the observed and randomized lags. The y axis represent cumulative proportion of responses, and the x axis the time intervals, which should be interpreted as seconds divided by 10, thus -3 sec to +3 sec.

| 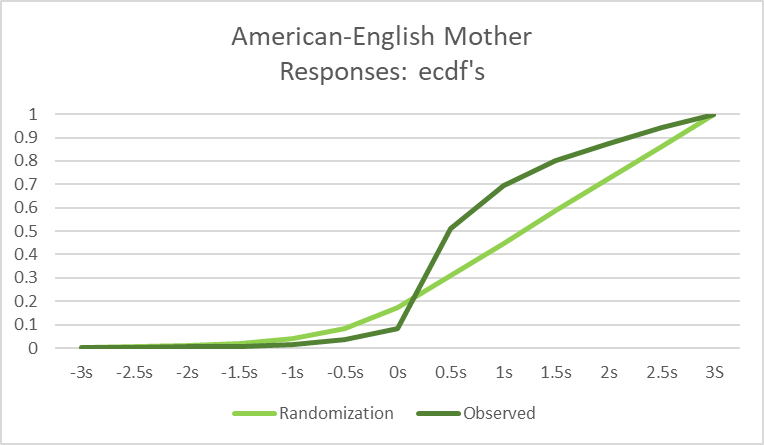 | 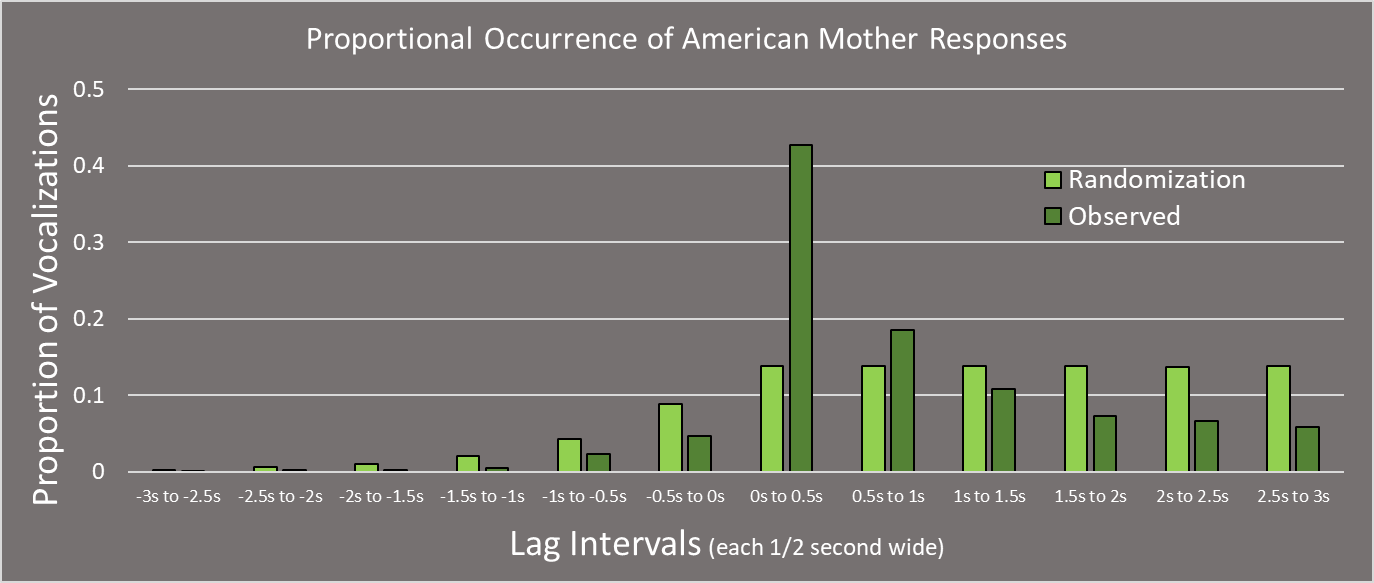 |
| --- | --- |
| **Figure C2** | |

**Figure C2: American Mothers Randomized Distribution**

The null hypothesis that the observed lags and the randomly generated lags came from the same distribution for American mothers was rejected with a p-value < 0.001 (test statistic = 0.25), see Figure C2.

| 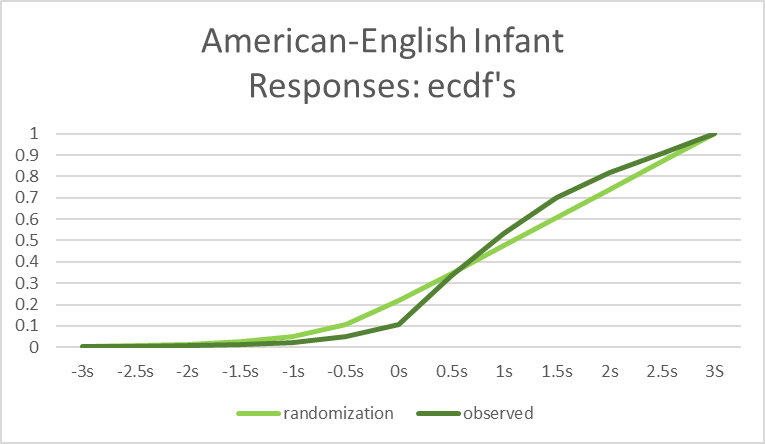 | 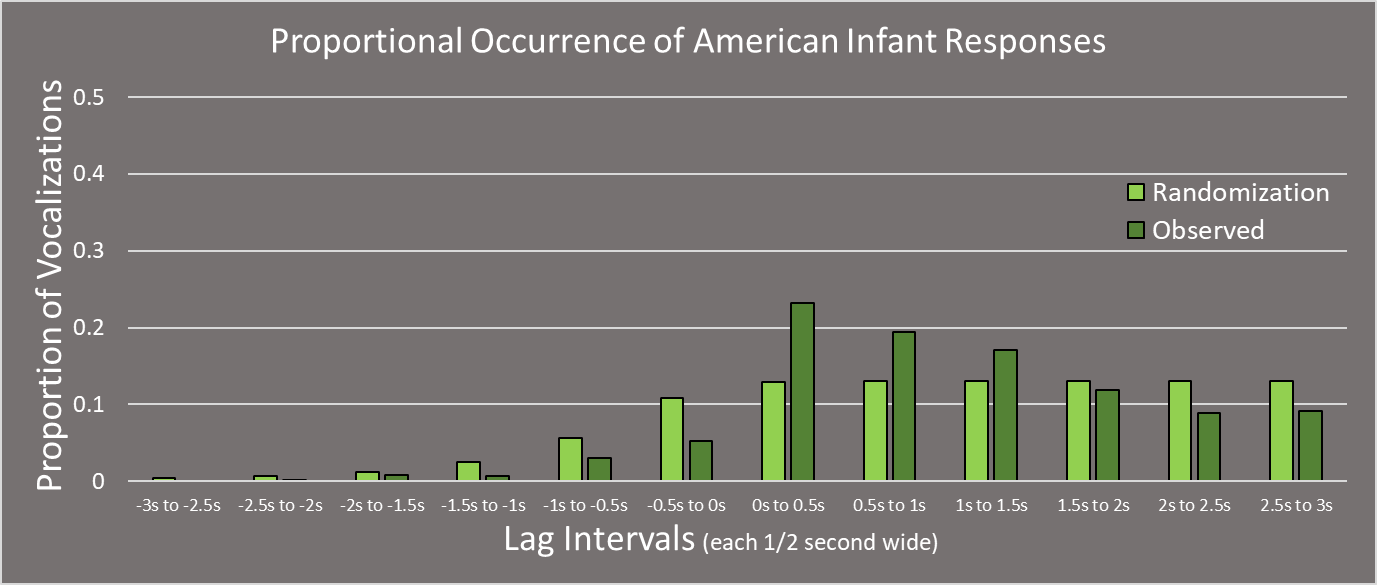 |
| --- | --- |
| **Figure C3** | |

**Figure C3: American Infants Randomized Distribution**

The null hypothesis that the observed lags and the randomly generated lags came from the same distribution for American infants was rejected with a p-value < 0.001 (test statistic = 0.11), see Figure C3.

**Empirical cumulative distribution functions of individuals by age group – number of lags available to be considered in each recording is in parenthesis.**

**Figure C4**

**Figure C4:** The x-axis in Figures C4 through C7 is labeled differently from the other figures in the main text and Supplementary Material. Each interval still represents .5 seconds, but each value has been multiplied by 10. So 5 means 0 to 0.5s, 10 means 0.5 to 1s, and so on. Similarly -5 means 0 to -0.5s, -10 means -0.5 to -1s, and so on. In Figure C4 it can be seen that 7 of the 9 Younger American English-learning infants showed rather similar patterns of responsivity to mother utterances, with ID9(1) being the most discrepant from the group, presumably because of having a very small number of responses that met the criteria specified for our analysis.

**Figure C5**

**Figure C5:** In Figure C5 it can be seen that 7 of the 10 Older American English-learning infants showed rather similar patterns of responsivity to mother utterances, with ID15(5), ID17(18) and ID6(2) being particularly discrepant from the group, ID6(2) presumably because of having a very small number of responses that met the criteria specified for our analysis.

**Figure C6**

**Figure C6:** In Figure C6 it can be seen that all of the 9 younger Lebanese Arabic-learning infants showed rather similar patterns of responsivity to mother utterances.

**Figure C7**

**Figure C7:** In Figure C7 it can be seen that 9 of the 10 older Lebanese Arabic-learning infants showed rather similar patterns of responsivity to mother utterances, with only ID18(5) being particularly discrepant from the group.
